# Knockout of Insulin-Like Growth Factor-1 Receptor Impairs Distal Lung Morphogenesis

**DOI:** 10.1371/journal.pone.0048071

**Published:** 2012-11-06

**Authors:** Ralph Epaud, Flore Aubey, Jie Xu, Zayna Chaker, Maud Clemessy, Alexandre Dautin, Karmène Ahamed, Monique Bonora, Nadia Hoyeau, Jean-François Fléjou, Arnaud Mailleux, Annick Clement, Alexandra Henrion-Caude, Martin Holzenberger

**Affiliations:** 1 INSERM UMRS 938, Hôpital Saint-Antoine, Paris, France; 2 UPMC, Université Paris 6, Paris, France; 3 INSERM U955, Faculté de Médecine, Université Paris-Est, Créteil, France; 4 APHP, Hôpital Saint Antoine, Paris, France; 5 INSERM UMR 700, Faculté Xavier Bichat, Paris, France; 6 APHP, Hôpital Trousseau, Paris, France; 7 INSERM UMRS 781, Hôpital Necker-Enfants Malades, Paris, France; University of Giessen Lung Center, Germany

## Abstract

**Background:**

Insulin-like growth factors (IGF-I and -II) are pleiotropic regulators of somatic growth and development in vertebrate species. Endocrine and paracrine effects of both hormones are mediated by a common IGF type 1 receptor (IGF-1R). Lethal respiratory failure in neonatal IGF-1R knockout mice suggested a particular role for this receptor in pulmonary development, and we therefore investigated the consequences of IGF-1R inactivation in lung tissue.

**Methods and Findings:**

We first generated compound heterozygous mutant mice harboring a hypomorphic (*Igf1r^neo^*) and a null (*Igf1r^−^*) allele. These IGF-1R^neo/−^ mice express only 22% of normal IGF-1R levels and are viable. In adult IGF-1R^neo/−^ mice, we assessed lung morphology and respiratory physiology and found normal histomorphometric characteristics and normal breathing response to hypercapnia. We then generated homozygous IGF-1R knockout mutants (IGF-1R^−/−^) and analyzed their lung development during late gestation using histomorphometric and immunohistochemical methods. IGF-1R^−/−^ embryos displayed severe lung hypoplasia and markedly underdeveloped diaphragms, leading to lethal neonatal respiratory distress. Importantly, IGF-1R^−/−^ lungs from late gestation embryos were four times smaller than control lungs and showed markedly thickened intersaccular mesenchyme, indicating strongly delayed lung maturation. Cell proliferation and apoptosis were significantly increased in IGF-1R^−/−^ lung tissue as compared with IGF-1R^+/+^ controls. Immunohistochemistry using pro-SP-C, NKX2-1, CD31 and vWF as markers revealed a delay in cell differentiation and arrest in the canalicular stage of prenatal respiratory organ development in IGF-1R^−/−^ mutant mice.

**Conclusions/Significance:**

We found that low levels of IGF-1R were sufficient to ensure normal lung development in mice. In contrast, complete absence of IGF-1R significantly delayed end-gestational lung maturation. Results indicate that IGF-1R plays essential roles in cell proliferation and timing of cell differentiation during fetal lung development.

## Introduction

Insulin-like growth factors (IGF-I and -II) control tissue homeostasis by regulating essential cell functions including proliferation, differentiation and survival, through their cognate tyrosine kinase receptor IGF-1R. IGF-II also interacts with a second receptor (M6P-R, or IGF-2R) that reduces IGF-II signaling through lysosomal degradation. During pre- and postnatal development and in the adult, IGF ligand and receptor expression are tightly regulated in a cell type-specific and spatiotemporal manner. Targeted mutation of IGF genes in the mouse showed that IGF signaling is relevant for development, homeostasis and repair of lung tissue [1]–[6]. Mutant mice completely lacking IGF-1R (IGF-1R^−/−^) reach only 45% of normal birth size, are unable to expand their lungs and die shortly after birth [1]. Similarly, mice lacking IGF-I are strongly growth retarded and show high postnatal mortality due to hypoplastic lungs marked by increased cellularity and collapsed alveoli [1], [2]. Prenatal lungs from IGF-I^−/−^ mice display abnormal cell proliferation, as well as altered alveolar epithelium and capillary differentiation [3]. IGF-II knockout mice, which show a less pronounced fetal growth deficiency, develop thickened pulmonary alveolar septa and altered alveolar organization [4]. IGF-1R mRNA expression is highest around embryonic day 18 (E18), and *ex vivo* stimulation of lung development by IGF-I and -II, shows that IGF signaling induces alveolar and vascular maturation in the late stages of fetal lung development [5]. Finally, IGF-1R signaling is also involved in vascularization and angiogenesis of human fetal lungs [7]. Recently, several heterozygous IGF-1R mutations have been identified in humans presenting with various degrees of intrauterine and postnatal growth retardation [8]–[12]. One patient with deletion of the distal long arm of chromosome 15, which includes the IGF-1R gene, was reported with lung hypoplasia [13]. These data are consistent with IGF-1R being an essential mediator of respiratory organ development.

Although IGF-1R knockout mice die from respiratory distress at birth, no study has so far focused on lung development in IGF-1R mutant mice. Here, we studied the role of IGF signaling in lung development and respiratory physiology using two different IGF-1R mutant models. First, we used IGF-1R knock-down mice (IGF-1R^neo/−^) [14], [15] that express only 22% of wild type IGF-1R levels in lung tissue, and that we found resistant to hyperoxia in a previous study [6]. In the present study, we showed that young adult IGF-1R^neo/−^ mice present with normal lung morphology and breathing. However, when we used mice with complete IGF-1R knockout (IGF-1R^−/−^), embryos showed conspicuous retardation of lung development, marked by increased cell proliferation and apoptosis.

## Results

### Young Adult IGF-1R^neo/−^ Mice Show Normal Lung Morphometry and Normal Lung Ventilation

**Figure 1:**
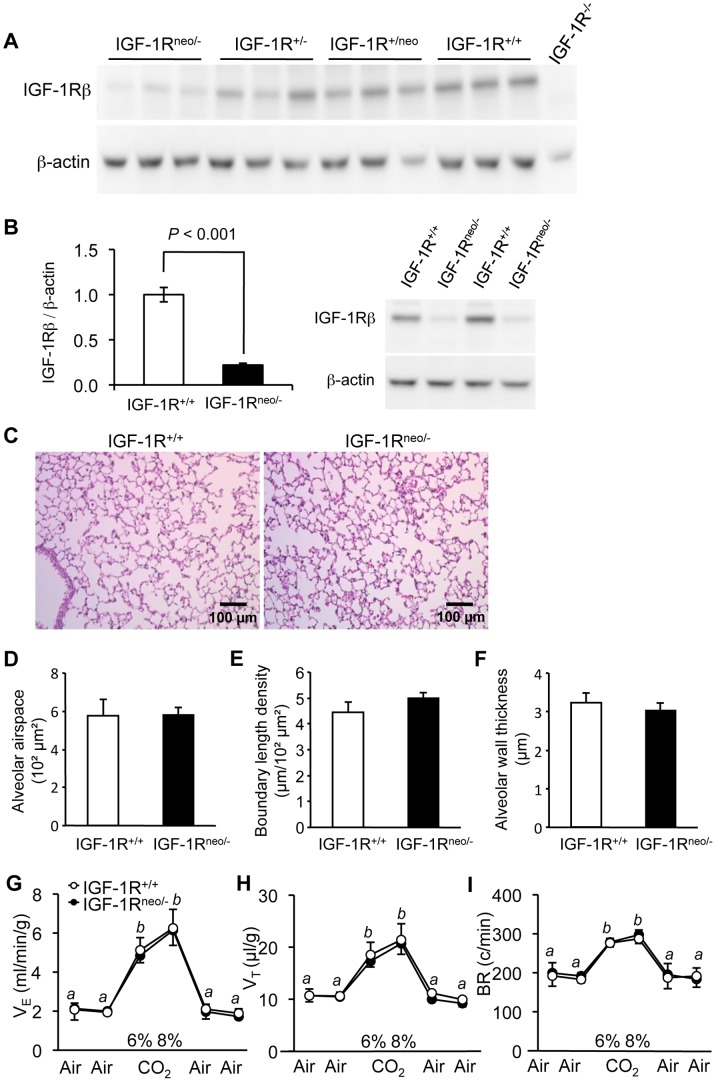
IGF-1R protein levels, lung histology and respiratory function in adult IGF-1R^neo/−^ mice. A , Western immunoblot of IGF-1R in lung from mice with distinct combinations of mutant IGF-1R alleles. Total proteins were extracted from lung tissue from IGF-1R^neo/−^, IGF-1R^+/−^, IGF-1R^+/neo^ and IGF-1R^+/+^ mice (n = 3 for each genotype), and IGF-1R^−/−^ embryo (negative control), and were probed with anti-IGF-1Rβ (upper panel) or anti-β-actin antibodies (lower panel). IGF-1R^neo/−^ mice have 22% of receptor levels present in IGF-1R^+/+^ mice (quantified in B), IGF-1R^+/−^ have 50%, IGF-1R^+/neo^ mice are between 70 and 80%, and IGF-1R^−/−^ mice lack IGF-1R completely. **B**, IGF-1R abundance determined in lung tissue. Bar graph shows IGF-1R levels relative to β-actin from 5 IGF-1R^+/+^ and 6 IGF-1R^neo/−^ individuals (Error bars SEM; Student’s *t*-test). Image shows 4 representative lanes from western immunoblot. **C**, Hematoxylin-eosin stained lung sections from IGF-1R^+/+^ and IGF-1R^neo/−^ males. **D**, Alveolar airspace, **E**, alveolar boundary length density, **F**, alveolar wall thickness, in IGF-1R^+/+^ (n = 4) and IGF-1R^neo/−^ mice (n = 4). Error bars indicate SEM; Wilcoxon Mann-Whitney U test. **G-I**, Respiratory function in adult IGF-1R^neo/−^ mice. Mice were challenged with 6% and 8% CO_2_. **G**, Minute ventilation (V_E_), **H**, tidal volume (V_T_), and **I**, respiratory frequency (BR) were measured in 6 individuals per group. Differences between room air and hypercapnia were significant, but no significant differences were found between genotypes. Values labeled *b* were different from *a* (*P*<0.005); Error bars indicate SEM; Wilcoxon Mann-Whitney U test.

In compound heterozygous IGF-1R^neo/−^ mice, IGF-1R expression is substantially decreased in all tissues. Using *in vitro* receptor ligand binding assay, we showed previously that IGF-1R^neo/−^ mice have four times less IGF binding sites than control mice in brain tissue [15]. Here we showed by western blot that in lung tissue, IGF-1R levels were diminished to 22% of control values (Fig. 1A, B). Nevertheless, comparing lung histology from 5-week-old IGF-1R^neo/−^ mice with control IGF-1R^+/+^ littermates, we found that lung architecture of IGF-1R^neo/−^ mice was not distinct from controls with respect to alveolar airspace, boundary length density and alveolar wall thickness (Fig. 1C-F). To assess respiratory function in these mutants, we recorded ventilation in conscious IGF-1R^neo/−^ and IGF-1R^+/+^ mice in ambient air and in response to hypercapnia. Baseline minute ventilation in ambient air was similar between groups (Fig. 1G). We then challenged the mice with 6% and 8% CO_2_, which markedly increased minute ventilation, tidal volume and breathing rate in both groups (Fig. 1H, I; *P*<0.005). However, the ventilatory responses to hypercapnia did not differ between IGF-1R^neo/−^ and IGF-1R^+/+^ mice. This suggested that low levels of IGF-1R in IGF-1R^neo/−^ mice are sufficient to ensure development of normal lungs and respiratory physiology.

### IGF-1R^−/−^ Embryos Display Pulmonary Hypoplasia with Delayed Saccular Development

In the absence of respiratory defects in IGF-1R^neo/−^ mice, we decided to study the effect of complete inactivation of IGF-1R on lung development using homozygous IGF-1R knockout (IGF-1R^−/−^) mice. At birth, IGF-1R^−/−^ mice showed skin color similar to controls. IGF-1R^−/−^ mutants moved limbs and trunks spontaneously and responded to tactile stimulation, similar to controls. However, within 15 min after birth, IGF-1R^−/−^ newborns exhibited cyanosis and agonal breathing, and died from acute hypoxia, as originally described by Liu *et al*. [1]. Whole lungs prepared from IGF-1R^−/−^ newborns did not float, indicating that they failed to inflate their lungs.

**Figure 2:**
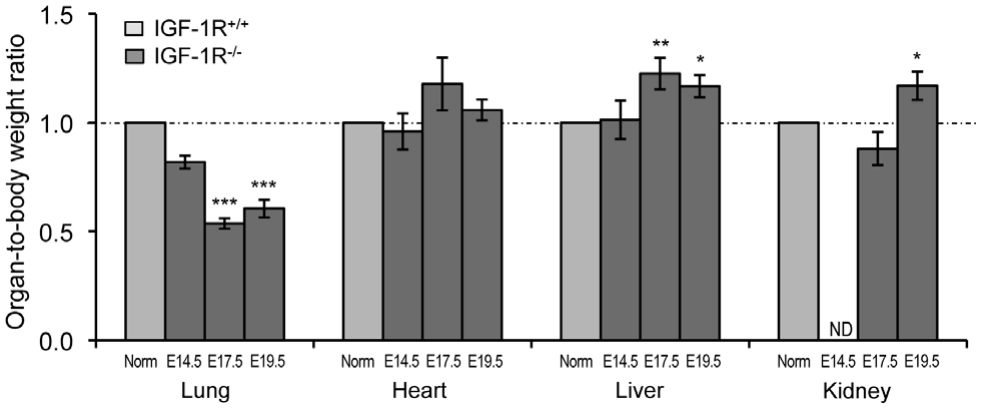
Growth retardation in IGF-1R^−/−^ embryos affects lung more than other tissue. Values represent organ weight relative to body weight (mean ± SEM), normalized to the stage-specific mean of the control (IGF-1R^+/+^) group. Organ/body weight ratio was calculated from data in Table 1. * *P*<0.05; ** *P*<0.01; *** *P*<0.001, compared with normalized IGF-1R^+/+^ data (Norm) of the same developmental stage; Student’s *t*-test; ND, not determined.

Next, we monitored lung development in the absence of IGF-1R during late embryonic life. We measured lung weight of IGF-1R^−/−^ and IGF-1R^+/+^ embryos at E14.5, E17.5 and E19.5, and compared with body weight and with organ weight of heart, liver and kidney. IGF-1R^−/−^ mice exhibited significant somatic growth retardation from E14.5 onwards and weighed 46% of controls at E19.5 (Table 1), similar to previous findings [1]. Lung weight was noticeably lower in IGF-1R^−/−^ embryos at E14.5, E17.5 and E19.5 (Table 1), with just 48%, 25% and 26% of the IGF-1R^+/+^ lung size, respectively. Importantly, unlike heart, liver and kidney, the ratio of lung to body weight was strongly decreased in IGF-1R^−/−^ embryos at E17.5 and E19.5 (Fig. 2), suggesting that organ hypoplasia was particularly pronounced in the lung, and that this developmental deficit occurred progressively around E14.5.

**Table 1:** Lung, heart, liver and kidney weight relative to body weight, in IGF-1R^+/+^ and IGF-1R^−/−^ embryos.

Developmental stage	E14.5		E17.5		E19.5	
Genotype	IGF-1R^+/+^	IGF-1R^−/−^	IGF-1R^+/+^	IGF-1R^−/−^	IGF-1R^+/+^	IGF-1R^−/−^
Body weight (mg)	184.4±12.3	130.2±2.8*	853.5±27.7	404.5±24.2***	1294.1±14.9	593.5±21.1***
Lung (mg)	3.26±0.10	1.58±0.03***	26.81±1.01	6.75±0.40***	40.76±1.57	10.73±0.82***
Heart (mg)	1.40±0.06	0.84±0.16**	4.95±0.27	2.81±0.35***	9.80±0.35	4.55±0.33***
Liver (mg)	12.30±0.89	9.62±0.83*	47.69±1.64	27.30±1.15***	73.49±3.30	38.52±1.95**
Kidney (mg)	0.50±0.05^a^	ND	7.50±0.27	3.11±0.34***	13.78±0.18	7.30±0.53***
Lung/BW (%)	1.49±0.12	1.22±0.04	3.14±0.03	1.68±0.08***	3.15±0.10	1.90±0.13***
Heart/BW (%)	0.78±0.05	0.74±0.06	0.58±0.02	0.68±0.07	0.76±0.03	0.80±0.04
Liver/BW (%)	6.26±0.23	6.35±0.56	5.60±0.13	6.86±0.40**	5.86±0.17	6.84±0.30*
Kidney/BW (%)	0.25±0.02^a^	ND	0.88±0.02	0.77±0.07	1.07±0.02	1.24±0.07*
N	7	6	13	7	8	6

Values are mean ± SEM. * *P*<0.05; ** *P*<0.01; *** *P*<0.001 compared with IGF-1R^+/+^ mice. Student’s *t*-test.

a n = 5; BW, body weight; ND, not determined.

**Figure 3:**
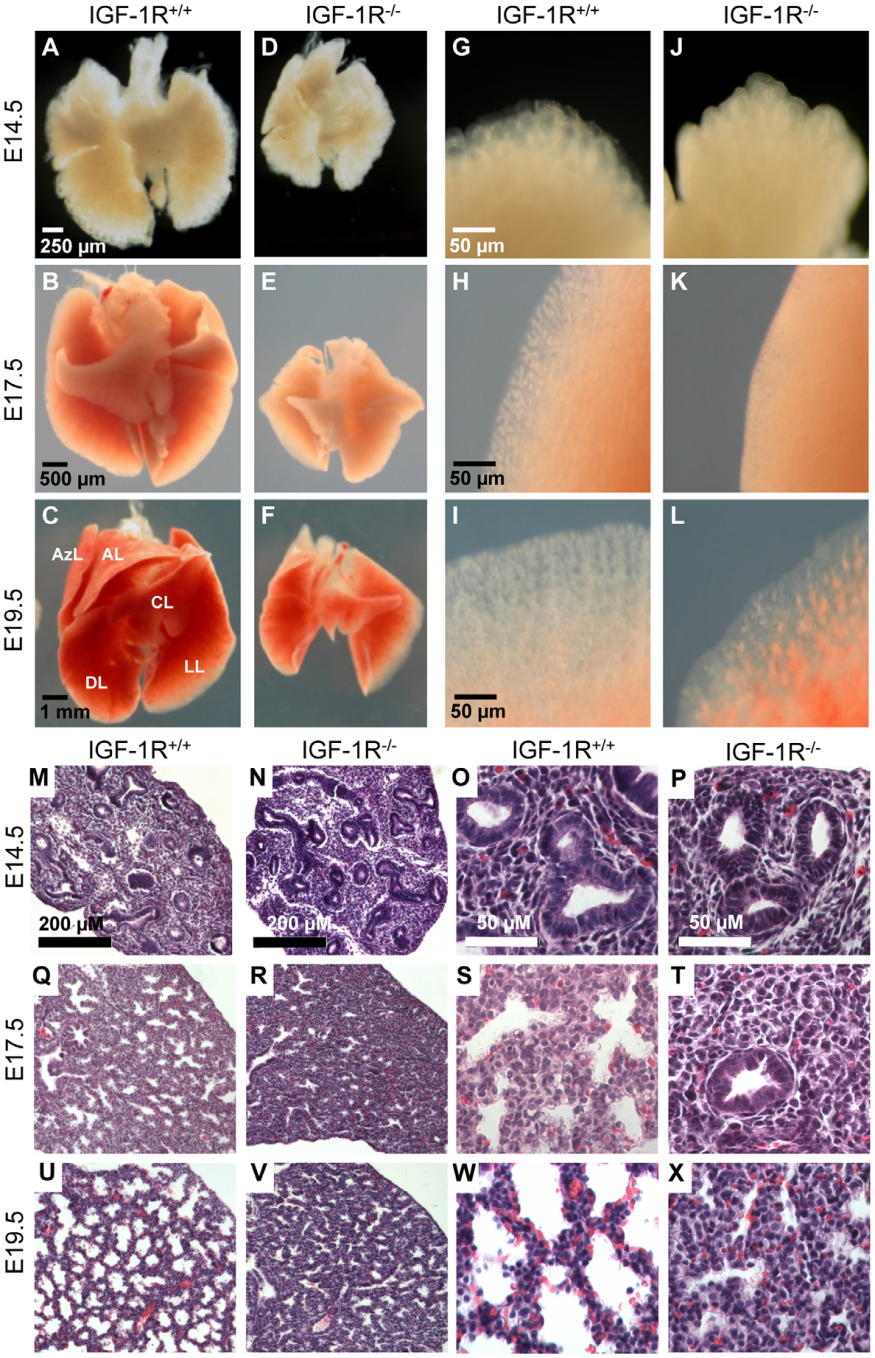
Lung development in late gestation IGF-1R^−/−^ mice. **A–L**, Lungs prepared from IGF-1R^+/+^ and IGF-1R^−/−^ embryos at developmental stages E14.5, E17.5 and E19.5. **A–F**, Ventral view of whole lungs. **G–L**, Rim of lung lobe. Abbreviations: AL, apical lobe; AzL, azygous lobe; CL, cardiac lobe; DL, diaphragmatic lobes; LL, left lobe. **M–X**, Lung histology of IGF-1R^+/+^ versus IGF-1R^−/−^ embryos. H&E stained lung sections at developmental stages E14.5 (**M**–**P**), E17.5 (**Q**–**T**) and E19.5 (**U**–**X**), showing that saccular walls are thicker and acinar buds smaller in IGF-1R^−/−^ embryos as compared with controls of the same stage. Note that histomorphological appearance is similar when comparing E19.5 IGF-1R^−/−^ (**V**, **X**) with two days younger E17.5 IGF-1R^+/+^ lungs (**Q**, **S**).

**Figure 4:**
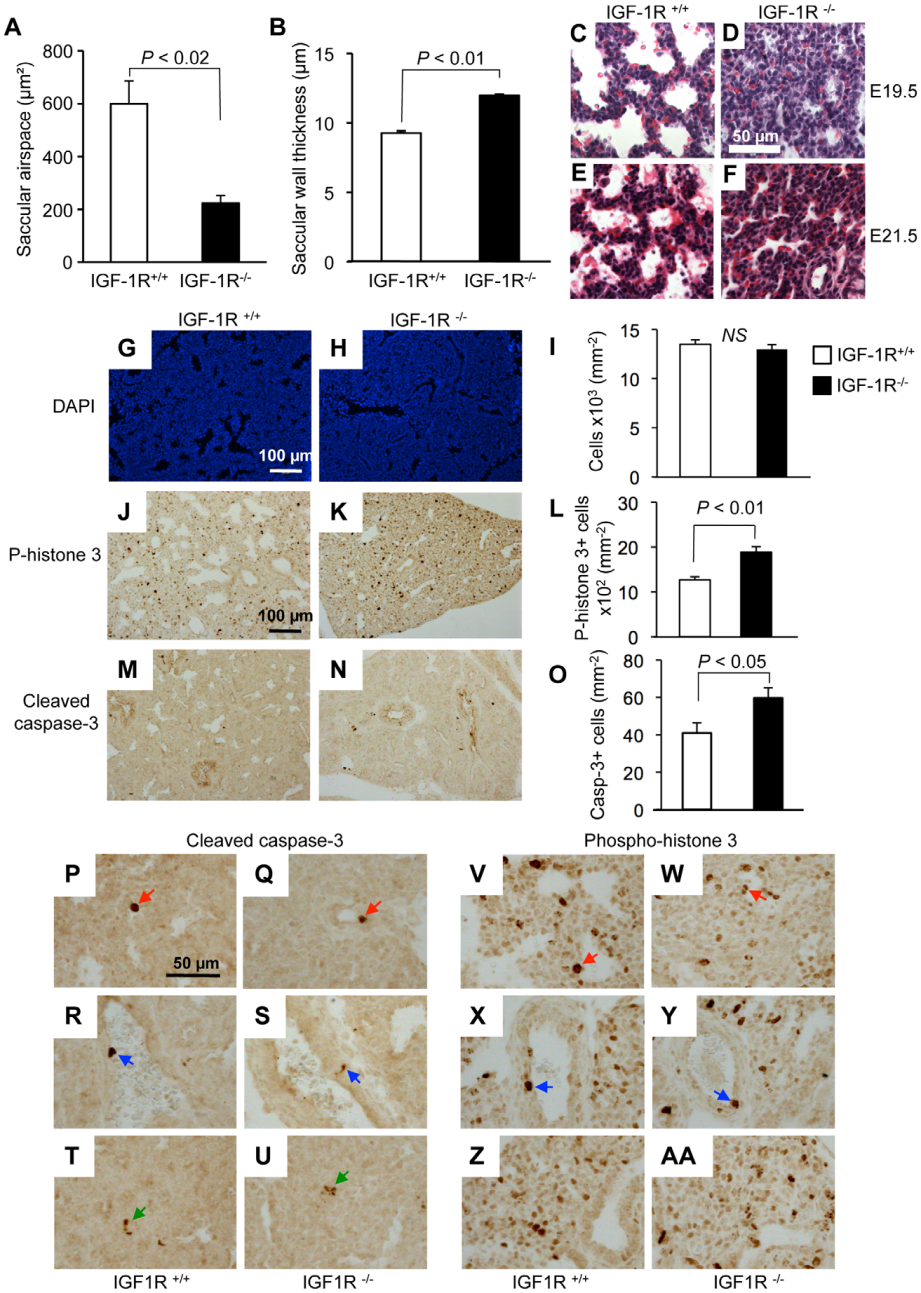
Lung histomorphology and cell turnover in the absence of IGF-1R. **A**, Saccular airspace and **B**, saccular wall thickness (mean ± SEM) at developmental stages E17.5 in IGF-1R^+/+^ (n = 4) and IGF-1R^−/−^ embryos (n = 4). Wilcoxon Mann-Whitney U test. **C–F,** Extended gestation period and lung histology. H&E stain of lung tissue from embryos at 19.5 (**C**, **D**) and 21.5 days (**E**, **F**). To extend gestation period up to 21.5 days, pregnant mothers were treated with progesterone from E17.5 onwards. Note the presence of red blood cell extravasation in E21.5 lung samples from IGF-1R^+/+^ and IGF-1R^−/−^ mice. **G–O,** Cell turnover in IGF-1R^−/−^ embryonic lung at E17.5. Lung histology from IGF-1R^+/+^ embryos (G, J and M) and IGF-1R^−/−^ embryos (H, K and N) at E17.5. Bar graphs (I, L and O) show quantification (mean ± SEM; n = 3–7 individuals per group; Student’s *t*-test). **G–I**, Cells were counted using DAPI staining (blue signal). **J–L**, Cell proliferation was measured using phospho–histone H3 immunohistochemistry (brown staining). **M–O**, Apoptosis was detected using cleaved caspase-3 immunohistochemistry (brown staining). Cleaved caspase-3 (P-U) and phospho-histone H3 labeling (V-AA) at high magnification showing examples for IHC-positive epithelial (red arrows), vascular endothelial (blue) and mesenchymal cells (green), as identified by their anatomical location. Note that many of the proliferating cells are located in areas that are composed of mostly mesenchymal cells.

Analysis of lung morphology showed normal anatomical organization of lobes in IGF-1R^−/−^ mutants (Fig. 3A-F), but higher magnification revealed a densification of the lung parenchyma in IGF-1R^−/−^ embryos (Fig. 3G–L). Histology of lung tissue showed conspicuous differences between IGF-1R^−/−^ and IGF-1R^+/+^ embryos, which were most prominent in the distal ducts (Fig. 3M–X). In IGF-1R^−/−^ embryos, the intersaccular mesenchyme was markedly thicker and the number of acinar buds lower as compared with controls, at E17.5 (Fig. 3Q–T) and at E19.5 (Fig. 3U–X). These characteristics were still similar in both genotypes at E14.5 (Fig. 3M–P). Together, this indicated that in the complete absence of IGF-1R, the normal physiological process of mesenchymal thinning was impaired, and suggested that lung development was delayed during the canalicular stage. Indeed, morphometry of lung tissue at E17.5 revealed that in lungs from IGF-1R^−/−^ embryos the saccular airspace was noticeably smaller (Fig. 4A) and the saccular wall thickness significantly increased (Fig. 4B), in accordance with the above histological observations. Using epithelial marker NKX2-1 (TTF-1), a transcription factor involved in lung organogenesis and epithelial differentiation, we determined the relative size of epithelial and mesenchymal compartments of lung parenchyma. This revealed that at E17.5, knockout lungs contained significantly less epithelium and less alveolar space than controls (epithelium: 50.1±6.8 *versus* 67.3±2.6%, *P*<0.05; alveolar space: 6.5±2.1 *versus* 13.1±1.3%, *P*<0.05; n = 3 and 5). In contrast, E17.5 knockout lung tissue harbored twice the amount of mesenchymal tissue compared with control lungs (43.4±9.0 *versus* 19.6±3.8%; n = 3 and 5, *P*<0.05).

To find out whether incomplete development of the lung and possibly also neonatal death can be rescued by prolonging the gestational period beyond full term, we treated pregnant females with progesterone and recovered E21.5 embryos by caesarian section. However, we found no evidence for catch up growth of the IGF-1R^−/−^ lungs during this period of extended gestation. Instead, lung development regressed when gestation was prolonged, in sharp contrast to heart and kidney that continued to increase organ weight (Table 2). Consistently, we found no histological evidence for lung maturation between E19.5 and E21.5 in IGF-1R^−/−^ embryos (Fig. 4C–F), and none of the E21.5 IGF-1R^−/−^ embryos was able to breathe.

**Table 2:** Effect of progesterone-induced extension of gestation on lung, heart, liver and kidney weight in IGF-1R^+/+^ and IGF-1R*^−/−^* embryos.

	IGF-1R^+/+^ a	ΔE19.5 [%] b	IGF-1R^−/−^ a	ΔE19.5 [%] b
Body Weight (mg)	1519.2±44.5	17	800.3±22.9***	35
Lung (mg)	34.78±0.95	**−15**	10.62±0.36***	**−1**
Heart (mg)	12.57±0.28	28	6.69±0.42***	47
Liver (mg)	51.20±1.57	**−**30	32.81±1.26***	**−**15
Kidney (mg)	16.97±0.99	23	14.77±0.47*	102
Lung/BW (%)	2.17±0.11	**−31**	1.34±0.05***	**−29**
Heart/BW (%)	0.84±0.01	11	0.82±0.04	2
Liver/BW (%)	3.24±0.07	**−**45	4.02±0.15**	**−**41
Kidney/BW (%)	1.12±0.06	5	1.84±0.02***	48
N	6		10	

Values are mean ± SEM. * *P*<0.05; ** *P*<0.01; *** *P*<0.001 compared with IGF-1R^+/+^ mice. Student’s *t*-test.

a Values at E21.5.

b ΔE19.5 indicates the difference compared with data from the same genotype at E19.5 (Table 1).

### Lung Hypoplasia in IGF-1R^−/−^ Embryos is Marked by Increased Cell Proliferation and Delayed Differentiation

**Figure 5:**
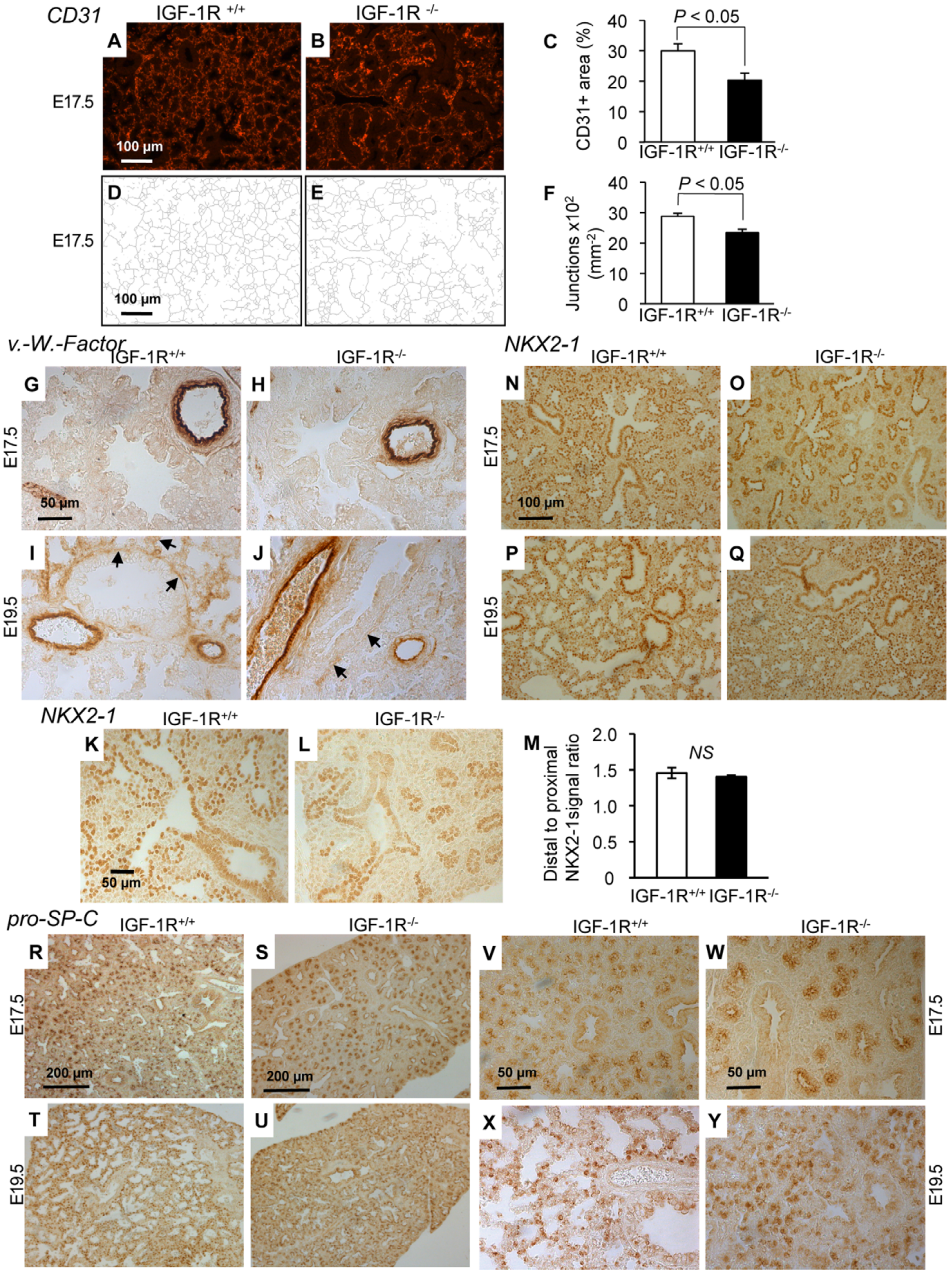
Immunohistochemistry of lung differentiation markers. **A** and **B,** Representative tissue sections from IGF-1R^+/+^ and IGF-1R^−/−^ embryos at stage E17.5 showing CD31-immunoreactivity specific for capillary endothelia. **C**, Morphometric comparison of CD31 signal between genotypes (n = 5 per group; two-tailed *t*-test). **D–F**, Capillary complexity was estimated calculating the density of capillary junctions from CD31 IHC. **G–J**, Sections from IGF-1R^+/+^ and IGF-1R^−/−^ embryos at E17.5 and E19.5 show IHC of blood vessel-specific von Willebrand protein. Arrows (I, J) point to small blood vessels developing in saccular walls. Large blood vessels were similarly marked in all specimen. **K–M**, Representative lung histology from IGF-1R^+/+^ and IGF-1R^−/−^ embryos at E17.5. NKX2-1 distal-to-proximal IHC signal ratio was measured in 6 IGF-1R^+/+^ and 5 IGF-1R^−/−^ embryos. NS, not significant; Wilcoxon Mann-Whitney U test. **N-Q**, Epithelial cell-specific NKX2-1 transcription factor was detected in IGF-1R^+/+^ and IGF-1R^−/−^ embryos at E17.5 and E19.5. **R–Y**, IHC of type 2-specific pro-SP-C at low (R-U) and high magnification (V-Y). Interestingly, for NKX2-1 and pro-SP-C, the IHC pattern of IGF-1R^−/−^ lungs at E19.5 resembles controls at E17.5 (panel N *versus* Q, R *versus* U, and V *versus* Y), suggesting an approximately 2-day developmental delay in IGF-1R^−/−^ end-gestational lungs.

To further investigate the role of IGF-1R during saccular stages of development, we assessed histoanatomy of embryonic lung tissue. At E17.5, cell density revealed by DAPI staining was similar in IGF-1R^−/−^ and IGF-1R^+/+^ embryos (Fig. 4G-I). Next, we assessed cell proliferation in IGF-1R*^−^* ^/*−*^ lungs using anti-phospho-histone H3 IHC, and found the number of proliferating cells significantly increased in IGF-1R^−/−^ lungs at E17.5 (1887±119 *versus* 1269±69, n = 3 and 5 per group, *P*<0.01; Fig. 4J-L). In addition, the percentage of cleaved caspase-3-positive lung cells at E17.5 was significantly higher in IGF-1R^−/−^ embryos (59.7±5.6 *versus* 41.0±5.5, n = 5 and 7 per group, *P*<0.05; Fig. 4M–O). Similar results were obtained using Ki67 and TUNEL staining (not shown). Proliferation and cell death concerned epithelial, vascular endothelial and mesenchymal cells (Fig. 4P-AA), but it was not clear whether IGF-1R inactivation affected all compartments to the same extent. However, since most of the proliferating cells superpose with mesenchyme, it can be deduced that cell turnover is increased also among mesenchymal cells. To investigate whether the delayed development in IGF-1R^−/−^ embryos was due to alterations in cell differentiation, we performed IHC on lung tissue from E17.5 and E19.5 IGF-1R^−/−^ and control embryos using cell type-specific markers of differentiation. We first assessed microvascular organization and capillary complexity at E17.5 using an antibody against CD31. We observed that the density of endothelial cells was significantly diminished in IGF-1R^−/−^ lung parenchyma compared with controls (Fig. 5A–C). Mutant tissue also showed a less developed microvascular network than controls, as revealed by the diminished number of capillary junctions (Fig. 5D–F). We then used an antibody against von Willebrand factor to monitor the development of blood vessels. At E17.5, smaller blood vessels were still scarce in both genotypes, while large blood vessels were strongly labeled in both knockout and controls (Fig. 5G, H). However, at E19.5, smaller blood vessels were clearly visible in controls while they just started appearing in IGF-1R^−/−^ lung tissue (arrows in Fig. 5I, J). We then evaluated epithelial expression of NKX2-1. At E17.5 we observed in both genotypes a distal-to-proximal difference of NKX2-1 expression between cuboidal cells of the acinar bud (distal) and columnar epithelial cells of the acinar tubule (proximal), with NKX2-1 being less abundant in the acinar tubule cells (Fig. 5K–M). This positive bud-to-tubule ratio was similar in IGF-1R^−/−^ and control mice. NKX2-1 IHC analysis demonstrated that epithelial cell organization changed profoundly in the transition between E17.5 and E19.5, in both genotypes (Fig. 5N–Q). Importantly, IGF-1R^−/−^ lungs displayed at E19.5 a distribution of NKX2-1 positive cuboidal epithelial cells that is typical for normal lung tissue at E17.5. Finally, to monitor type 2-cell differentiation, we analyzed IHC of surfactant protein pro-SP-C. Again, IGF-1R^−/−^ tissue exhibited at E19.5 a differentiation pattern that normal (IGF-1R^+/+^) lungs already showed at E17.5 (Fig. 5R–Y). Taken together, analysis of cell markers demonstrated that major differentiation processes in the developing lung parenchyma of IGF-1R^−/−^ mice were significantly delayed and in fact arrested in the canalicular stage. Collectively, our data suggest a key role for IGF-1R in regulating proliferation, apoptosis and timing of differentiation in developing lung tissue. Increased proliferation with increased apoptosis and incomplete epithelial differentiation are hallmarks of late canalicular stages, and results presented here clearly illustrate the substantial delay in lung maturation prevailing in the late stages of IGF-1R knockout embryogenesis.

### Development of the Diaphragm is Markedly Affected in IGF-1R^−/−^ Embryos

**Figure 6:**
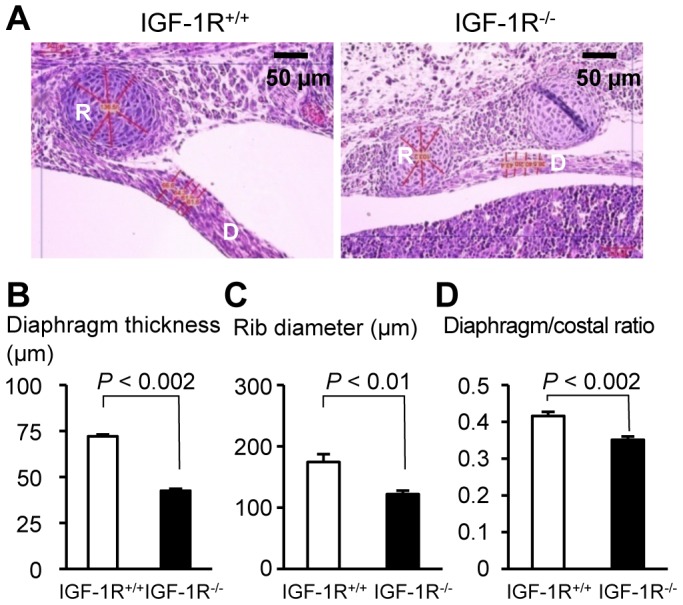
Development of diaphragm and chest in the absence of IGF-1R. **A**, Hematoxylin-eosin stained transversal section of thoracic wall and diaphragm in control (left) and IGF-1R^−/−^ embryos (right) at E17.5. Bar graphs compare **B**, diaphragm thickness, **C**, rib diameter, and **D**, diaphragm-to-rib ratio (mean ± SEM) in IGF-1R^+/+^ embryos (n = 4) and IGF-1R^−/−^ embryos (n = 4). R, Rib; D, diaphragm. Wilcoxon Mann-Whitney U test.

With respect to possible extra-pulmonary causes of delayed lung development, we noticed that the diaphragm of IGF-1R^−/−^ embryos, although intact across the abdominal cavity and with no evidence for hernia, was significantly thinner than that of IGF-1R^+/+^ embryos, as shown in transverse sections of the trunk (42.5±0.9 *versus* 72.1±2.4 µm, *P*<0.002; Fig. 6A, B). At the same time, rib diameter was relatively less affected in IGF-1R^−/−^ embryos (122±6 *versus* 174±13 µm, *P*<0.01; Fig. 6C), such that the diaphragm/rib ratio was significantly smaller in IGF-1R^−/−^ embryos as compared with IGF-1R^+/+^ (0.35±0.01 *versus* 0.42±0.01, *P*<0.002; Fig. 6D). This suggested an over-proportional reduction in IGF-1R^−/−^ diaphragm muscle mass.

## Discussion

The aim of this study was to examine the consequences of IGF-1R inactivation on lung development. We first investigated the effects of a substantial reduction of receptor levels in the lungs of IGF-1R^neo/−^ mice on respiratory physiology and morphology. Although IGF-1R^neo/−^ mice displayed significant growth retardation at birth and thereafter, we observed no evidence for respiratory distress. Likewise, histomorphology at embryonic stages (data not shown) and at adult stages was not different between IGF-1R^neo/−^ and IGF-1R^+/+^ mice. Moreover, the normal ventilatory response to hypercapnia observed in IGF-1R^neo/−^ mice was indicative of intact respiratory physiology, and we conclude that as little as 22% of wild type IGF-1R protein levels are sufficient to ensure normal lung development and function. These findings are consistent with observations in humans, where heterozygous mutation of IGF-1R is associated with delayed growth and sometimes with retarded mental development, but rarely with altered respiratory function [13].

Liu *et al*. [1] and Holzenberger *et al.* [14] reported a generalized organ hypoplasia in IGF-1R^−/−^ embryos. Here, we focused on lung development and observed pulmonary hypoplasia in IGF-1R^−/−^ embryos as early as E14.5, a phenotype that is in line with the pulmonary hypoplasia observed in IGF-I and IGF-II gene knockout mice. IGF-I and IGF-II both act through IGF-1R, and deletion of IGF-I or IGF-II genes results in severe embryonic growth retardation, affecting all organs and tissues [1], [16], [17]. These mice suffer from lung and muscle hypoplasia, which explains their respiratory distress and high mortality [1], [4], [16]. Here we showed that IGF-1R knockout affected in particular the lungs, suggesting that the IGF system plays an exceptionally strong role in the development of fetal lung.

The main consequence of complete IGF-1R inactivation for mouse lung development is failure of progression from canalicular to saccular structures and increased proliferation in perinatal fetuses (stages E17.5 to E19.5). Our data showed that IGF-1R^−/−^ lungs at E17.5 are extremely hypoplastic and retained in the pseudoglandular stage. Eventually, by E19.5, the lungs of the IGF-1R^−/−^ mice had moved through the canalicular stage and were transitioning into the early saccular stage, but were still severely hypoplastic. Importantly, thinning of the alveolar septa is necessary for subsequent perinatal maturation and development of efficient gas exchange of the lungs. In fact, compared with the control mice at E19.5, IGF-1R^−/−^ lungs exhibited thickened primary alveolar septa, which may be the principal cause of neonatal respiratory failure. Immunostaining of pro-SP-C, NKX2-1, CD31 and von Willebrand factor demonstrated that major differentiation processes were delayed in the developing lung parenchyma of IGF-1R^−/−^ mice. Meanwhile, our study did not reveal any significant difference between genotypes in the number of NKX2-1 positive proximal and distal cuboidal lung epithelial cells, nor in the distal-to-proximal ratio of NKX2-1 expression that may have suggested an altered pattern of alveolar maturation.

Paradoxically, the progressive lung hypoplasia observed in end-gestation IGF-1R^−/−^ mice was associated with increased cell proliferation. In normal lungs, the mitotic rate drops and differentiation prevails before birth. We reasoned that sustained high proliferation rates observed in the IGF-1R^−/−^ mice could result from retarded pulmonary differentiation. Moreover, loss of IGF-1R signaling must cause either increased cell death or shifted cell fate choices that could explain the lack of airway expansion. Delayed pattern of cell differentiation together with increased apoptosis indicates that both processes are indeed altered. The generalized mitotic activity observed in prenatal IGF-1R^−/−^ mice may contribute to diminished airway space and alveolar collapse, which is similar to the findings in IGF-I deficient lungs [3]. Lung hypoplasia observed in IGF-1R^−/−^ mice at E17.5 was accompanied by an increase in cell death that can also be explained by the fact that the structural remodeling of IGF-1R^−/−^ lungs is not complete, so that mutant lungs might be retained in earlier stages of lung development. As we showed here, these earlier developmental stages are marked by a dominant mesenchymal compartment, less epithelial structures and smaller alveolar spaces. Since many of the proliferating cells reside in the mesenchymal compartment, part of the observed increase in cell turnover in mutants can be explained by developmental differences in tissue composition. Alternatively, anti-apoptotic effects of IGF-1R signaling have been demonstrated in immature lungs, where more than 20% of interstitial fibroblasts undergo apoptosis after the period of bulk alveolarization, resulting in a substantial reduction in interstitial volume [18]. IGF-1R mRNA expression and protein levels were both down-regulated in lipid-filled interstitial fibroblasts (LIF) after alveolar formation on postnatal days 16 to 18, suggesting a role for IGF-1R in lung fibroblast survival [18]. Thus, lack of IGF signaling could explain increased apoptosis and reduced lung size in mutants. On a more speculative note, one could imagine that the impaired differentiation process resulting from the loss of IGF-1R may lead to reduced fluid secretion into the developing airways, or interfere with continuous drainage of airway fluid into the amniotic space. Clearly more data on pathophysiology and gene expression during late developmental stages are needed in these mutants.

We and others reported previously that intercostal muscles were drastically underdeveloped in IGF-1R^−/−^ mice [1], [15]. Severe muscle hypoplasia in thoracic muscles may be responsible for a decrease in fetal breathing movements and thereby cause secondary lung hypoplasia. In fact, several clinical case reports indicate that infants without fetal breathing movements *in utero* suffer from newborn pulmonary hypoplasia, and rarely survive the neonatal period [19], [20]. Similarly, knockout of myogenin, a major regulator of skeletal muscle differentiation, leads to marked defects in skeletal muscle development, in particular retarded fiber development, but also abnormal lung morphology. As myogenin is only expressed in skeletal muscle, but not in lung tissue, it was postulated that lung hypoplasia in that model is secondary to abnormal development of skeletal muscle [21]. These observations suggest that diaphragm hypoplasia observed in IGF-1R^−/−^ mice may account for a significant part of the pulmonary hypoplasia phenotype and the delayed saccular development. A combination of direct effects (due to lack of IGF-1R in lung tissue) and indirect effects (secondary to marked muscle hypoplasia) could explain the exceptionally strong developmental delay observed in IGF-1R^−/−^ lungs.

In conclusion, we found that lung development progresses to completion in knock-down IGF-1R^neo/−^ mutants, demonstrating that partial IGF-1R inactivation is well tolerated. Complete IGF-1R inactivation, in contrast, produced severe delay in lung maturation *in utero* leading to lung hypoplasia and entailing neonatal death. Delayed lung development in end-gestational IGF-1R^−/−^ embryos was characterized by high cell proliferation and incomplete differentiation that are the hallmarks of the canalicular stage of lung development.

## Materials and Methods

### Mice

All experiments were conducted according to the European Communities Council Directive (86/609/EEC) for the care and use of animals for experimental procedures and complied with the regulations of the *Comité d’Ethique pour l’Expérimentation Animale* ‘Charles Darwin’, registered at the *Comité National de Réflexion Ethique sur l’Expérimentation Animale* (Ile-de-France, Paris, N°5). All experiments were supervised by MH (agreement no. 75-444 to MH, specifically approved by the *Direction des Services Vétérinaires*, Paris, France). All efforts were made to minimize suffering.

We used IGF receptor null (*Igf1r^−^*) and knock-down (Igf1r^neo^) alleles described previously [14], [6], [22]. Both alleles were maintained in 129/Sv genetic background. For studies in embryos, IGF-1R^+/−^ males were mated to 9 to 12-week old IGF-1R^+/−^ females. The morning of vaginal plug was defined as day 0.5 of embryonic development (E0.5). Litters were sacrificed at E14.5, E17.5 and E19.5, and embryonic stages were confirmed using tables of normal mouse development [23]. For developmental studies, we used embryos of both sexes, since no sex-differences of lung development *in utero* were observed among IGF-1R^−/−^ or IGF-1R^+/+^ (control) mice. To prolong gestation up to 21.5 d, we injected females with 1 mg progesterone s.c. every 12 h from E17 onwards. Progesterone (Sigma) was suspended in ethanol at 100 mg/mL and diluted to 10 mg/mL in sterile sunflower oil. Embryos were recovered at E21.5 by caesarian section. Genotyping was performed by PCR as described [6]. Litters showed Mendelian proportion of mutant allele combinations. For studies in young adult mice, IGF-1R^+/−^ males were mated to 9 to 12-week old IGF-1R^neo/+^ females. Mice lived under SPF conditions in individually ventilated filter-cages.

### Macroscopic Analysis of Lungs and Preparation of Tissues

The uterus of pregnant females was removed under deep anesthesia and embryos prepared eliminating all extra-embryonic tissues. Embryos were immediately weighed on a fine balance, sacrificed and skin biopsies taken for genotyping. Heart, liver, kidneys and lungs were removed and weighed. Lungs were observed using a Leica MZ125 with SPOT v3.2.0 digital camera (4x to 100x magnification) and fixed in 4% paraformaldehyde, dehydrated and embedded in paraffin.

### Western Immunoblot

Lung parenchymal tissue (100 mg) was homogenized and incubated for 30 min in ice-cold extraction buffer (50 mM Tris pH 7.4, 150 mM NaCl, 2 mM EDTA, 5 mM DTT, 0.3% NP-40, 0.2% Triton X-100 and Complete-Mini protease inhibitor; Roche, Meylan, France). Homogenates were centrifuged and protein concentration determined in supernatants using the Bradford method. Proteins (20 µg) were resolved by 12% SDS-PAGE and electro-transferred to PVDF membranes (Millipore, Billerica, MA). Membranes were blocked, the upper half (containing IGF-1R) probed ON at 4°C with primary anti-IGF-1Rβ antibody (1∶4000; Cell Signaling #3027, Beverly, MA) and subsequently incubated for 60 min at room temperature (RT) with a horseradish peroxidase-labeled secondary goat anti-rabbit antibody (1∶5000; Invitrogen 65–6120, Carlsbad, CA). The lower half of the membranes was incubated overnight (ON) at 4°C with primary mouse anti-β-actin antibody (1∶70,000; Sigma A5316) and then incubated for 60 min at RT with a horseradish peroxidase-labeled secondary goat anti-mouse antibody (1∶5000; Invitrogen 62–6520). Blots were developed using Novex chemiluminescence substrate (Invitrogen WP20005) and analyzed using a ChemiDoc apparatus and Quantity One 4.2.1 software (Bio-Rad Laboratories, Hercules, CA).

### Immunohistochemistry

For immunohistochemistry and hematoxylin-eosin (H&E) staining, 7 µm tissue sections were used, deparaffinized in xylene and rehydrated in decreasing concentrations of ethanol. Microwave antigen retrieval was performed for NKX2-1 with 10 mM citrate buffer (pH 6) for 15 min. Endogenous peroxidase was inactivated with 3% H_2_O_2_ in PBS for 40 min, and sections incubated in 3% normal goat serum (NGS) for 30 min. We used rabbit anti-cleaved caspase-3 antibody (#9661, 1∶200, Cell Signaling; Antigen retrieval with 1 mmol EDTA), rabbit anti-phospho-histone H3 antibody (#06–570, 1∶200, Millipore; Antigen retrieval with citrate pH6), anti-von Willebrand factor antibody (A0082, 1∶500; Dakocytomation, Trappes, France), anti-NKX2-1 (anti-TTF1) antibody (WRAB-TTF1, 1∶5000; Seven Hills Bioreagents, Cincinnati, OH) and anti-pro-SP-C (WRAB-SPC, 1∶4000; Seven Hills Bioreagents) primary antibodies diluted in PBS with 1% NGS (1 h at RT or ON at 4°C). Sections were then incubated for 30 min with biotinylated goat anti-rabbit IgG (BA-1000, 1∶200; Vector Laboratories, Burlingame, CA). Antigen-antibody complexes were amplified with ABC-peroxidase complex (PK-6100, Vectastain ABC Elite kit, Vector Laboratories) and revealed with DAB+ peroxidase substrate kit (K3468, Dako). Control sections were incubated with a pre-immune rabbit IgG and showed no specific staining. We also used rat anti-CD31 antibody (#553370, 1∶50, BD Biosciences; Antigen retrieval with trypsin buffer) for immunofluorescence microscopy. Anti-CD31 antibody was incubated overnight at 4°C. Secondary antibody (#A10522, Invitrogene, 1∶100) was incubated for 2 h at RT. For blocking we used TNB buffer (Tris pH 7.6, NaCl) with 3% Donkey serum.

### Morphometry

#### Adult mice

Lung morphometry was performed in IGF-1R^neo/−^ and IGF-1R^+/+^ mice as described [24]–[26]. Mice were anesthetized, chest opened after exsanguination, trachea exposed and lungs perfused *in situ* with 10% neutral-buffered formalin. Under constant pressure of 500 Pa the trachea was ligated, lungs excised and placed in fixative for 5 d prior to paraffin embedding. Serial 4 µm sections of both lungs were stained with H&E. Sections with maximum cross-section of parenchyma were selected for morphometry using digitized image analysis [27]. Micrographs were captured using a Laborlux D Leitz (Leica SA, Rueil Malmaison, France) with monocad Sony videocamera, and analyzed with Biocom (Biocom Imaging Division, Les Ulis, France). Digital images were filtered, binarized, and segmented into background and structures of interest. Total length of tissue-air-interface, representing the alveolar boundary length, was measured in 12 different 5×10^5^ mm^2^ non-overlapping parenchymal fields. We selected areas devoid of large conductive airways, arteries, or veins from 4 different tissue sections. Measurements evaluated the alveolar boundary length density expressed per unit of parenchymal surface. Mean alveolar airspace was determined from the sum of the lumen divided by the number of identified alveoli. Thickness of the alveolar walls was determined from >10 linear measurements per field.

#### Embryos

Thickness of diaphragm and diameter of the tenth rib were measured at E19.5 in frontal sections of the chest in 4 individuals per genotype. Saccular wall thickness and airspace were measured in E17.5 embryos (4 individuals per group) using on average 20 measurements per mouse. To determine the proportion of epithelial, mesenchymal and vascular cell compartments in lung parenchyma (expressed as relative surface area), representative 7 µm sections stained for NKX2-1 or CD31were analyzed using ImageJ software (version 1.45, NIH, Bethesda, MD). Phospho-histone H3 and cleaved caspase-3 positive cells were counted in three representative microscope views from three different tissue sections per individual. The most apical and basal regions, and the area close to the lung hilus were not considered. All cell counts were performed using 400x magnification. For CD31, the surface area occupied by stained cells was determined using ImageJ software. For DAPI cell count, micrographs were analyzed using ImageJ algorithms for segmentation. For NKX2-1 morphometry, we compared immunostaining in cuboidal epithelial cells of acinar buds (distal ducts) with staining in columnar epithelial cells of acinar tubules (proximal ducts) using sections from 5 IGF-1R^−/−^ and 6 IGF-1R^+/+^ mice. All slides were processed in one experiment as a single batch. Micrographs were taken with 40x objective using a Leica DM5000B microscope equipped with DFC300FX CCD camera. Care was taken not to saturate the signal. To avoid that potential differences in NKX2-1 expression between apical and basal lung influence the results, we chose regions that were not apical or basal. Since the branching generation of bronchioles influences NKX2-1 expression, we considered only the recent acinar tubules and buds in the outer zone of the lung. We excluded structures located adjacent to the lung surface, at the rim of the tissue sections, where specific IHC signal could overlay with staining artifacts. For each mouse, we analyzed a minimum of 10 structures, consisting of acinar tubule and adjacent bud, from three different tissue sections. In each pair of acinar bud and tubule identified, NKX2-1 signal intensity was quantified from at least 10 epithelial cells per structure using ImageJ software. Results were averaged and compared between bud and tubule cells. From these data we computed the average ratio for each individual.

### Respiratory Physiology

Experiments were carried out on 6 IGF-1R^neo/−^ and 6 IGF-1R^+/+^ young adult males. Ventilatory parameters were recorded in conscious, unrestrained mice placed in a whole-body plethysmograph using the barometric method [28]. The pressure resulting from breathing was detected using a transducer (Validine DP103/12; Validine, Northridge, CA) connected to animal and reference chamber (400 mL each). Spirograms were recorded using respiratory data acquisition software (CIO-DAS 1602/16 interface and ELPHY software). Calibration was performed prior to experiments by repeated injections of 50 µL air into the chamber. Animals were weighed and placed into the chamber. A thermistal probe (BIO-BIT14, Bioseb, Chaville, France) was inserted rectally and secured at the base of the tail and the chamber. Measurements were made during air breathing every 5 min during a 20-min period, to obtain baseline values. Subsequently, the animals were exposed to two levels of hypercapnia (6% and 8% CO_2_), each challenge lasting for 10 min. Mice were allowed to recover in normoxia for 20 min. Minute ventilation (V_E_), tidal volume (V_T_) and breathing rate (BR) were determined. For each 5-min recording, values were averaged on 50 to 100 contiguous breaths.

### Statistical Analysis

Groups of mice were compared using the Wilcoxon Mann-Whitney U test (SAS software, version 9.1 SAS Institute, Cary, NC, USA) or Student’s *t*-test, as indicated in figure legends. Bar graphs represent mean ± SEM. *P*<0.05 was considered significant.
